# Autoimmune Cholangitis: A Variant Syndrome of Autoimmune Hepatitis

**DOI:** 10.1155/2014/501530

**Published:** 2014-09-21

**Authors:** Brij Sharma, Sujeet Raina, Rajesh Sharma

**Affiliations:** ^1^Department of Gastroenterology, Indira Gandhi Medical College, Shimla, Himachal Pradesh 171001, India; ^2^Department of Medicine, Dr. Rajendra Prasad Government Medical College, Tanda, Kangra, Himachal Pradesh 176001, India

## Abstract

Autoimmune cholangitis (AIC) or autoimmune cholangiopathy is a chronic inflammation of liver and a variant syndrome of autoimmune hepatitis (AIH). We present a case of an adult female who had biochemical features of cholestasis and transaminasemia but aminotransferases were not in the hepatitis range and had histological evidence of bile duct injury which was subsequently diagnosed as autoimmune cholangitis.

## 1. Introduction

Autoimmune cholangitis (AIC) or autoimmune cholangiopathy is a chronic inflammation of liver and a variant syndrome of autoimmune hepatitis (AIH). It is an “outlier” (findings that are inconsistent with the definite diagnosis of AIH) rather than an “overlap” (features of AIH and another liver disease) syndrome. Clinical features including fatigue and pruritus and laboratory features of cholestasis are present. Serological findings are high titers of antinuclear antibodies (ANA) and/or smooth muscle antibodies (SMA). Antimitochondrial antibodies (AMA) are undetectable. Histopathology of liver tissue reveals bile duct injury in the form of cholangitis and ductopenia with little or no portal inflammation. The diagnosis implies the absence of inflammatory bowel disease and/or a normal cholangiogram [1]. We present a case of an adult female who had biochemical features of cholestasis and transaminasemia but aminotransferases were not in the hepatitis range and histological evidence of bile duct injury which was subsequently diagnosed as AIC. The case is presented as it is a rare variant form of AIH; there is paucity of such cases in literature and overall experience with this condition remains relatively small and anecdotal.

## 2. Case History

A 53-year-old female was admitted with history of jaundice for last 2 months. There was no history of prodrome of viral hepatitis, no history suggestive of cholestasis features, and no history of drug intake, fever, GI bleed, and pain abdomen. Appetite was normal. Review of other systems was normal. No similar complaints in the past were present. There was no history of any comorbidity and addiction and appetite was normal. On general physical examination icterus was present. Liver was palpable 8 cm below costal margin and span was 15 cm. Liver was firm and nontender. Rest of the examination was normal.

**Figure 1:**
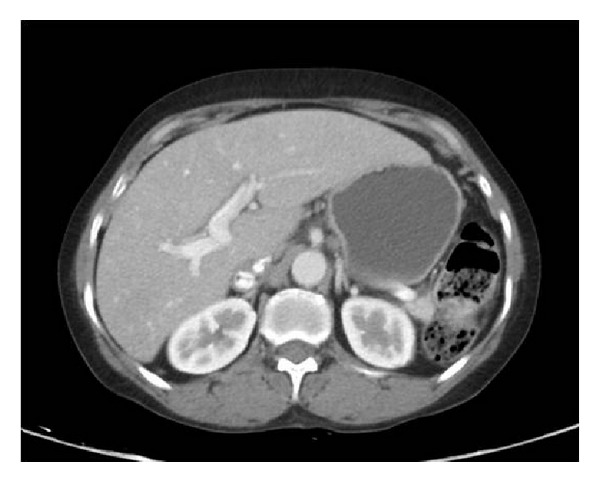
CT of abdomen showing hepatomegaly.

**Figure 2:**
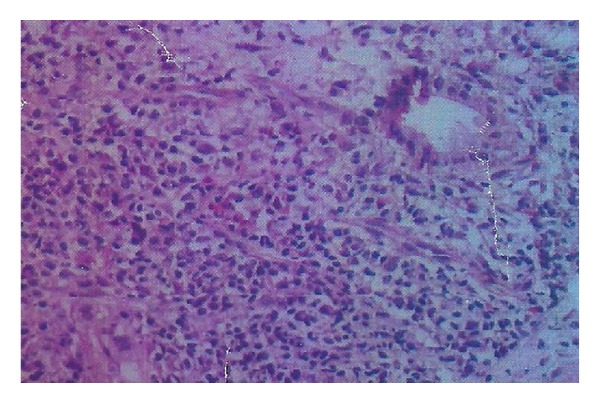
Photomicrograph showing chronic inflammatory infiltrate and portal to portal bridging fibrosis (×400, H&E).

**Figure 3:**
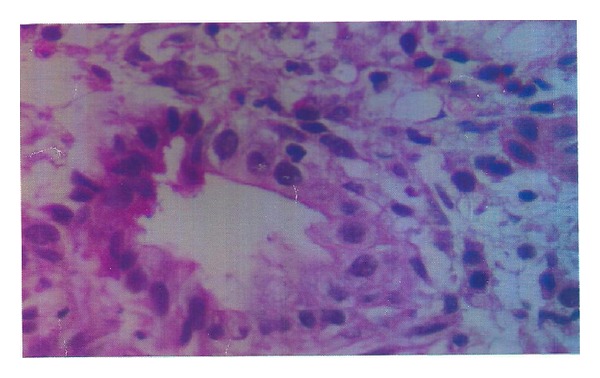
Photomicrograph showing biliary ductal infiltration by inflammatory cells with destruction of bile ducts and periductular collection of plasma cells, lymphoid cells, and histiocytes. Foam cell change in hepatocytes is observed (×1000, H&E).

Her investigations were hemoglobin: 10.5 gm%; total leukocyte count: 6390/cmm; Platelets: 1.6 lac/cmm; total protein: 9.4 gm/dL; albumin: 3.1 gm/dL; total bilirubin: 3.3 mg/dL; direct bilirubin: 1.7 mg/dL; serum alkaline phosphatase: 1378 U/L; serum aspartate aminotransferase: 128 U/L; serum alanine aminotransferase: 90 U/L. Renal functions, creatine phosphokinase (CPK), prothrombin time, lactate dehydrogenase (LDH), angiotensin converting enzyme (ACE) levels, reticulocyte count, and thyroid functions were normal. Virology markers like HBsAg, anti-HCV, HBV DNA, HCV qualitative, IgM anti-HEV, and IgM anti-HAV were negative. In the serological markers, rheumatoid factor, IgA anti-tTG antibodies, antismooth muscle antibodies (ASMA), and antimitochondrial antibodies (AMA) were negative. Antinuclear antibodies (ANA) were found positive against liver-kidney-microsomes (LKM) and IgG4 levels were normal. Upper GI endoscopy was normal. CT of abdomen revealed hepatomegaly and normal portal vein (Figure 1). Liver biopsy showed chronic inflammatory cell infiltrate including plasma cells in portal triads (Figure 2). Biliary ductal infiltration by inflammatory cells with destruction of bile ducts and periductular collection of plasma cells, lymphoid cells, and histiocytes forming florid bile ductal lesions were observed (Figure 3). Ductopenia was present. Portal to portal bridging fibrosis was evident (Figure 2). Hepatocytes showed occasional confluent necrosis with moderate interface hepatitis. Stain for copper showed focal positivity in less than one-third of periportal hepatocytes. Based on clinical presentation, biochemical, serology, and virology reports, and histopathology, possibility of autoimmune cholangitis was made. Patient was treated with ursodeoxycholic acid and aziothioprine (2 mg/kg bodyweight). Liver functions tests returned to normal and hepatomegaly regressed during followup.

## 3. Discussion

The name “immune cholangitis” was introduced first by Brunner and Klinge to describe a condition seen in three women (two were mother and daughter) who had liver disease which clinically, biochemically, and histologically seemed to be typical of primary biliary cirrhosis, except that the serum antimitochondrial antibody (AMA) test was negative in all three; all three were antinuclear antibody (ANA) positive. The patients described showed a favorable clinical response to immunosuppressive therapy (azathioprine and prednisone) [2]. In 1993 Ben-Ari et al. described four patients with features overlapping those of primary biliary cirrhosis and autoimmune chronic active hepatitis. These patients were ANA and SMA positive and were AMA negative and termed to have* autoimmune cholangiopathy* [3]. In 1997 Heathcote coined the phrase “autoimmune cholangitis” to describe AMA negative primary biliary cirrhosis [4].

Since the first description, several cases with similar histological and clinical features have been reported and referred to as primary autoimmune cholangitis or autoimmune cholangiopathy [5].

Patients with AIH may present atypically and have features associated with another liver disease, that is, “overlap syndromes” or with findings that are inconsistent with the criteria of definite diagnosis of AIH, that is, “outlier syndromes”. Overlap syndromes include combinations of AIH and PBC, AIH and PSC, AIH and chronic viral hepatitis and AIC [1].

Patients with AIC are AMA negative and often present with serum ANA and/or ASMA. Several studies support the view that AIC and PBC are variants of one single disease only differing in serum autoantibody pattern [6]. Autoimmune cholangitis is probably a heterogeneous syndrome that includes patients with AMA-negative PBC, small-duct PSC, AIH with bile duct damage, concurrent AIH and small duct PSC, and various transition states [7]. Prospective studies have emphasized the inability to classify these individuals into a single diagnostic category. AIC shares many features with PBC and is therefore also called AMA-negative PBC. Like PBC, it is characterized by a female preponderance and a cholestasis serum enzyme pattern and it slowly progresses to fibrosis and cirrhosis of the liver if left untreated [4].

Histological features of cholangitis are portal inflammation with bile duct injury, including ductopenia, and copper stains of hepatic tissue may be positive and indicative of chronic cholestasis [1, 5]. Features of hepatocellular damage such as piecemeal necrosis, confluent necrosis, and spotty lobular necrosis are rare in AIC. Histology could not differentiate AIC from PBC and it was concluded that it might be a subtype of AMA-negative PBC that occurs at a younger age and shows lesser fibrosis than PBC [5]. Histological findings may be indistinguishable from PBC or PSC, and individual patients cannot be discriminated by clinical, laboratory, genetic, or histological parameters [7].

Treatment is empiric and consists of immunosuppressors (aziothioprine or prednisone), ursodeoxycholic acid (UDCA), or a combination of both. Responsiveness to corticosteroids or UDCA is variable and generally poor. Indeed, most studies emphasize an inability to induce histological improvement with either drug. Therapy should be reserved mainly for those individuals who are symptomatic with jaundice, pruritus, and/or malaise [1].

## 4. Conclusion

Standardization of diagnostic criteria for overlap syndromes has not been achieved so far, since these disorders are uncommon. It remains unclear whether these overlap syndromes form distinct disease entities or are only variants of the major immune hepatopathies.
